# C-reactive protein as a predictor of mortality in tuberculosis: systematic review and meta-analysis

**DOI:** 10.1186/s12879-026-13462-9

**Published:** 2026-05-06

**Authors:** Jessica Edelyne, Muhammad Prasetio Wardoyo, Luthfiyah Zanida Putri, Salsabila Hulwani, Assica Permata Amalya Hakiman, R. Muhammad Kevin Baswara, Yogik Onky Silvana Wijaya, Erlina Burhan

**Affiliations:** 1 Master in Biomedical Science, Faculty of Medicine, Public Health, and Nursing, Universitas Gadjah Mada, Yogyakarta, Indonesia; 2 Faculty of Pharmacy, Université de Montpellier, Montpellier, France; 3 Respiratory Programmatic Implementation and Research Institute, Jakarta, Indonesia; 4 Mahidol University, Bangkok, Thailand; 5 Clinical Trials Unit, University College London, London, England, UK; 6 Faculty of Medicine, Universitas Indonesia, Jakarta, Indonesia; 7 Department of Biochemistry, Faculty of Medicine, Public Health, and Nursing, Universitas Gadjah Mada, Yogyakarta, Indonesia; 8 Department of Pulmonology and Respiratory Medicine, Faculty of Medicine, Universitas Indonesia, Jakarta, Indonesia; 9 Persahabatan Hospital, Jakarta, Indonesia

**Keywords:** C-reactive protein, Tuberculosis, Mortality, Biomarker, Prognosis

## Abstract

**Background:**

Tuberculosis (TB) remains a leading cause of global mortality, underscoring the need for accessible prognostic tools. C-reactive protein (CRP) is a widely available acute-phase biomarker that may predict outcomes in TB. This systematic review and meta-analysis assessed the prognostic value of baseline CRP in predicting mortality among adults with TB.

**Methods:**

Eligible studies included cohort studies, observational studies, and control arms of randomized controlled trials involving adults (≥ 18 years) with microbiologically confirmed TB. The exposure of interest was baseline CRP level, and the primary outcome was mortality, reported as adjusted hazard ratios (aHRs) or odds ratios (aORs). Data extraction followed the CHARMS-PF checklist. Risk of bias was assessed using the QUIPS, while certainty of evidence was evaluated using GRADE.

**Results:**

From 1,277 records, nine studies met the inclusion criteria (three retrospective cohorts, three prospective cohorts, and three case-control studies) conducted in Japan, China, South Korea, Chinese Taipei, and South Africa. Five studies reported significant associations between elevated baseline CRP and increased mortality. In pooled analyses, three studies reporting aORs showed a modest but statistically significant association between higher baseline CRP and mortality (aOR 1.07, 95% CI 1.03–1.11; I²=0%). In contrast, pooled aHRs from four studies (aHR 1.02, 95% CI 0.99–1.05; I²=60%) and pooled cHRs from two studies (cHR 1.75, 95% CI 0.58–5.29; I²=91%) were not statistically significant.

**Conclusions:**

Baseline CRP showed a modest association with mortality in pooled aOR analyses, but not in pooled hazard ratio analyses. Nevertheless, its low cost and wide availability suggest potential utility as part of multimodal prognostic models, especially in high-risk populations and resource-limited settings. High-quality prospective studies with standardized CRP protocols are needed to clarify its prognostic role.

**Registration:**

PROSPERO (CRD420251101984).

**Supplementary Information:**

The online version contains supplementary material available at 10.1186/s12879-026-13462-9.

## Introduction

Tuberculosis (TB) remains a major global health challenge, with substantial morbidity and mortality. In 2023, TB caused an estimated 1.25 million deaths, making it the leading cause of death from a single infectious agent, surpassing COVID-19 [1]. Despite effective antituberculosis regimens, treatment outcomes vary widely, from full recovery to death or chronic post-TB lung disease [1, 2]. These variations highlight the role of host and immunological factors in TB pathophysiology. Understanding these factors may enable prognostic profiling and early identification of patients who could benefit from intensified or host-directed therapies [3].

Biomarkers offer potential as prognostic indicators, helping clinicians identify high-risk patients and guide targeted treatment strategies. However, no well-established biomarker currently reflects host status or reliably predicts TB outcomes, limiting individualized care [4]. The need for a reliable, accessible prognostic marker is therefore urgent to support early intervention and improve survival.

C-reactive protein (CRP), an acute-phase protein produced by the liver during systemic inflammation, has emerged as a promising host-derived biomarker in TB [5, 6]. Although CRP is elevated in other infections, such as pneumonia, which may coexist with TB, it retains potential value when measured at diagnosis [5]. Elevated CRP concentrations have been observed in both pulmonary and extrapulmonary TB, including spinal disease, and levels typically decline following successful treatment [7, 8]. In critically ill patients with pulmonary TB, higher baseline CRP has been associated with markers of disease severity, including higher APACHE II scores and lower albumin levels, suggesting that it may reflect early clinical deterioration [9]. Importantly, CRP testing is inexpensive and widely available, making it feasible in resource-limited settings [5].

The growing evidence base has led the WHO to recommend CRP measurement for TB screening. WHO recommends CRP for TB screening among people living with HIV, with a cut-off of > 5 mg/L preferred because of its higher sensitivity, while higher cut-offs such as > 10 mg/L provide greater specificity [10]. These recommendations highlight the need to clarify the prognostic value of CRP across diverse patient groups, including HIV and non-HIV populations, and pulmonary and extrapulmonary TB.

Given the wide availability and low cost of CRP testing, clarifying its prognostic value in tuberculosis remains clinically relevant. However, it remains uncertain whether baseline CRP is independently associated with mortality across different TB populations and clinical settings. This systematic review and meta-analysis aim to (1) synthesize current evidence on CRP as a prognostic biomarker in adult TB patients, and (2) assess its association with mortality in terms of both relative and absolute effect estimate.

## Methods

### Protocol and registration

This systematic review and meta-analysis followed the Preferred Reporting Items for Systematic Reviews and Meta-Analyses (PRISMA) 2020 guidelines, as provided in Appendix 1 and 2 [11]. The protocol was prospectively registered in the International Prospective Register of Systematic Reviews (PROSPERO; registration number CRD420251101984) [12].

### Search strategy and selection criteria

A comprehensive literature search was conducted on July 10, 2025, across the Cochrane Library, PubMed, Scopus, MEDLINE via Ovid, ProQuest, and medRxiv. The search was based on our PICO framework. The population was adult patients aged 18 years and older diagnosed with tuberculosis. The exposure was higher baseline C-reactive protein (CRP) level measured at the time of TB diagnosis. The control was normal/Lower baseline C-reactive protein (CRP) level measured at the time of TB diagnosis. The outcome was mortality, regardless of the cause of death. The strategy combined free-text keywords and controlled vocabulary (MeSH/Emtree) terms related to tuberculosis, C-reactive protein, prognosis, and mortality. Search terms included “tuberculosis,” “TB,” “C-reactive protein,” “CRP,” “mortality,” “death,” and “prognosis.” The search covered inception to June 2025 and was restricted to English-language full-text articles. A detailed strategy is provided in Appendix 3.

Eligible studies included prospective or retrospective cohorts, case-control studies, and randomized controlled trials (RCTs) arms involving adults (≥ 18 years) with microbiologically confirmed pulmonary or extrapulmonary TB. Studies were required to report baseline CRP levels at diagnosis and provide mortality outcomes expressed as hazard ratios, odds ratios, or relative risks. Studies limited to latent TB were excluded.

### Study selection and data extraction

Two reviewers independently screened all retrieved records, with discrepancies resolved through consensus with a third reviewer. Duplicate records were removed using the Rayyan software [13]. Following title and abstract screening, three reviewers (JE, LZP, SH) assessed full texts for eligibility. Disagreements were resolved by two additional reviewers (MPW, APAH) or by group discussion until consensus was reached.

Data extraction was performed independently by five reviewers (JE, LZP, SH, MPW, APAH) using the CHARMS-PF checklist for prognostic factor studies. Extracted information included study design, setting, sample size, mortality rates, type of TB, follow-up duration, inclusion-exclusion criteria, comorbidities, sociodemographic data, CRP measurements and cut-offs, and effect estimates.

We extracted reported effect estimates for the association between CRP and mortality directly from each included study, including crude hazard ratios (cHRs), adjusted hazard ratios (aHRs), and adjusted odds ratios (aORs), together with their corresponding 95% confidence intervals (CIs). Effect estimates were recorded separately according to whether they were crude or adjusted. No hazard ratios were reconstructed from Kaplan-Meier curves or calculated from raw individual-level data.

Although the protocol initially stated that study authors would not be contacted, this was reconsidered during the review. Authors of overlapping studies were contacted to clarify participant duplication and request adjusted effect estimates. However, only one responded, and no additional usable data were obtained.

### Quality assessment

Risk of bias was assessed using the Quality in Prognosis Studies (QUIPS) as it was a tool specifically made to assess the risk of bias in prognostic studies. QUIPS domains (study participation, prognostic factor measurement, outcome measurement, confounding, and analysis) were rated as low, moderate, or high risk of bias [14, 15].

Certainty of evidence was further evaluated using the Grading of Recommendations Assessment, Development, and Evaluation (GRADE) approach [16]. Publication bias was to be assessed through funnel plots or Egger’s test if minimum included studies of 10 were achieved; if this minimum included studies was not achieved, publication bias assessment wouldn’t be done due to reliable statistical testing [17]. Quality assessments were performed independently by a minimum of two researchers, and any disagreements were resolved through in-depth discussions by all researchers (JE, LZP, SH, MPW, APAH) until an agreement was reached.

### Data synthesis and analysis

Data were synthesized to evaluate CRP as a prognostic biomarker for TB mortality. Statistical analyses were performed using Review Manager (RevMan) version 5.4 (Cochrane Collaboration). A random-effects model was applied to account for between-study heterogeneity. Because the included studies reported different effect measures, meta-analyses were performed separately for aORs, cHRs, and aHRs. Heterogeneity was assessed visually using forest plots and quantified with the I² statistic. Subgroup analysis was to be conducted whenever two studies with similar subgroup characteristics and similar measure of effect size were available. Several subgroup characteristics considered were specific age-based population, HIV status, TB subtype, and type of CRP value reported (continuous vs. dichotomous). Meanwhile, sensitivity analysis was conducted in the event of high heterogeneity due to methodological consideration, such as low sample size and difference in CRP measurement method.

## Results

### Study flow and characteristics

**Fig. 1:**
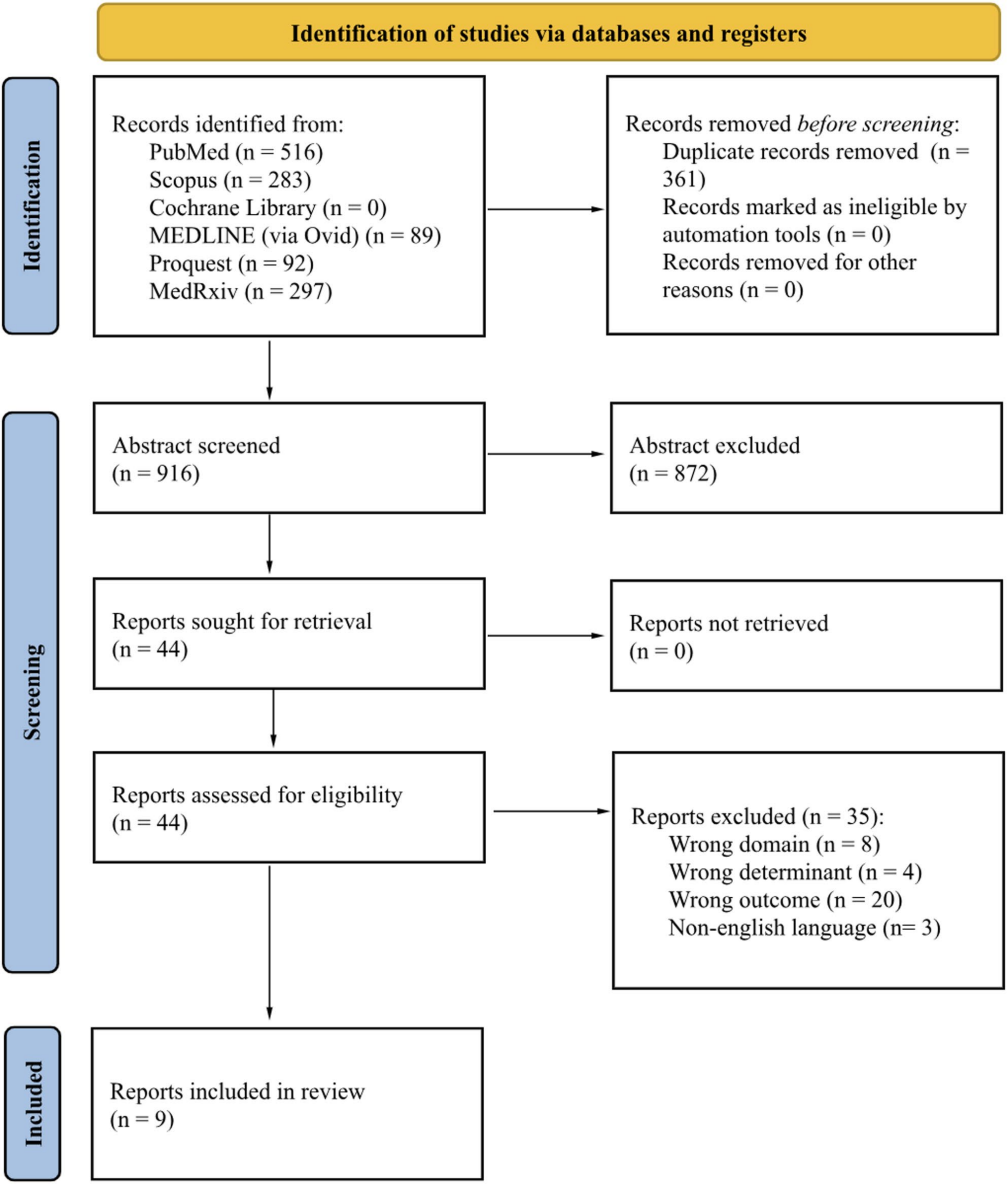
Literature searching and selection process

The initial search retrieved 1,277 records across the selected databases. After deduplication in Rayyan, 361 records were removed, leaving 916 for title and abstract screening. Of these, 872 were excluded for the following reasons: inappropriate study design (e.g., case reports, reviews, conference abstracts), wrong population (e.g., pediatric studies or mixed pediatric-adult data without separate analyses), wrong determinant (studies not assessing CRP), wrong domain (studies on diseases other than tuberculosis), wrong outcome (no mortality data or no quantitative CRP effect estimates such as hazard ratios or odds ratios), and non-English language. After title and abstract screening, 44 reports were sought for retrieval. All 44 reports were successfully retrieved and assessed for eligibility. Of these, 35 reports were excluded after full-text assessment for predefined reasons, leaving nine studies for inclusion in the systematic review and meta-analysis. The reasons for exclusion of assessed studies were provided in Appendix 4. Ultimately, nine studies met the eligibility criteria and were included in the systematic review and meta-analysis. The study selection process is illustrated in Fig. 1, while main characteristics of the included studies are summarized in Table 1.

**Table 1:** The characteristics of the included studies

First Author (Year)	Country	Study Design	Population	Sample Size (*N*)	CRP as a Dichotomized indicator?	Type of mortality	Effect Estimate	Key Findings
Cudahy (2018) [18]	South Africa	Prospective cohort	HIV-positive MDR-TB	20	No	16-week mortality, cause not specified	aHR 2.94 (1.03–8.35); AUC 0.74; Sens 100%, Spec 71%	Persistently high CRP linked to death; 100% sensitivity for < 44% decline by W2
Honjo (2020) [19]	Japan	Case control	Elderly, bacteriologically-diagnosed, pulmonary TB patients	275	No	TB-related in-hospital mortality	cHR 1.076 (1.026–1.127), *p* = 0.002 (univ); aHR 0.990 (0.916–1.070), *p* = 0.804 (multiv)	CRP is significant in univariate analysis, not multivariate analysis.
Huang (2014) [20]	Chinese Taipei	Prospective cohort	Adult culture-confirmed pulmonary TB patients	243	Yes (≥ 2.38 mg/L)	All-cause 6-month mortality	cHR 3.25 (1.65–6.42); AUC 0.75 (0.67–0.83)	CRP predicted mortality; not significant in multivariate analysis
Kan (2019) [21]	Japan	Retrospective cohort	Smear-positive, pulmonary TB patients	123	No	All-cause in-hospital mortality	cHR 1.046 (0.997–1.097), *p* = 0.064	CRP associated with death in univariate analysis, not multivariate
Kim et al. (2012) [22]	South Korea	Case Control	Adult bacteriologically-confirmed pulmonary TB patients	269	No	All-cause mortality	aOR 1.62 (95% CI: 0.38–6.95); *p* = 0.517	CRP is significant in univariate analysis; it is not identified in multivariate analysis.
Kobayashi (2024) [23]	Japan	Case-control	Adult TB patients, regardless of anatomical location	244	No	In-hospital all-cause mortality	aHR 1.04 (1.01–1.08), *p* = 0.007	CRP is independently associated with mortality
Komiya (2020) [24]	Japan	Retrospective cohort	Elderly, bacteriologically-diagnosed, pulmonary TB patients	185	Yes (≥ 10 mg/L vs. < 5 mg/L)	All-cause mortality during treatment	aOR 1.057 (1.014–1.101), *p* = 0.009	Associated with death during treatment
Liu (2020) [9]	China	Retrospective cohort	Adult pulmonary TB patients accompanied with acute respiratory failure	153	No	In-hospital mortality, cause not specified	aOR 1.012 (1.004–1.019), *p* = 0.003; AUC 0.818; Sens 83.3%, Spec 73.1%	High CRP independently predicted mortality
Wang (2019) [25]	China	Prospective cohort	Adult culture-confirmed pulmonary TB patients	287	No	All-cause 12-month mortality	aOR 1.10 (1.02–1.33), *p* = 0.038	CRP is associated with mortality in both univariate and multivariate

The review incorporated nine observational studies published between 2012 and 2024, all involving adult TB patients. Studies were conducted in five countries: Japan (*n* = 4) [18–21], China (*n* = 2) [9, 22], South Korea (*n* = 1) [23], Chinese Taipei (*n* = 1) [24], and the South Africa (*n* = 1) [25]. Study designs included three retrospective cohorts, three prospective cohorts, and three case-control studies. All participants were aged ≥ 18 years. Most included studies focused on hospitalized patients and involved pulmonary or predominantly pulmonary TB populations, while one study included adults with TB regardless of anatomical location and one focused on HIV-positive multidrug-resistant TB. Mortality was consistently reported as the primary outcome, defined as all-cause death, although follow-up duration varied across studies.

Three studies from Japan [18, 19, 21] were identified as having overlapping populations based on study location and period. Nonetheless, all were retained because they applied different inclusion criteria (e.g., age restriction, ICU admission, co-infection status) and provided distinct quantitative estimates. In the meta-analysis, all three were included, as they reported different effect measures: aHR, cHR, and aOR, respectively.

### Critical appraisal: risk of bias, certainty assessment, and publication bias

Methodological quality was assessed using the QUIPS tool, while certainty of evidence was evaluated with the GRADE approach. Risk of bias assessment using the QUIPS tool indicated a generally low risk across all six domains (study participation, prognostic factor measurement, outcome measurement, study confounding, statistical analysis and reporting, and attrition). Moderate risk was noted in Cudahy (2018) for study participation and in Liu (2020) for statistical reporting.

In contrast, the GRADE assessment revealed lower certainty of evidence: six studies were rated as very low certainty and three as low certainty. Consequently, despite consistent trends in study findings, the overall confidence in the estimated effects remains limited. Publication bias cannot be assessed with funnel plots or Egger’s test, given the small number of included studies (*n* = 9). A detailed summary of the appraisal is provided in Appendix 5.

### Study findings

The median patient age varied across included studies. For example, Komiya (2020) reported a median age of 82 years (IQR 79–88) among elderly patients [21], while Kobayashi (2024) reported a median age of 77 years (IQR 68–84) [20]. Mortality outcomes also varied, including in-hospital, six-month, twelve-month, all-cause, and TB-specific mortality, which introduced heterogeneity in the synthesis of findings.

Five studies reported statistically significant associations between higher baseline CRP levels and increased mortality in multivariate analyses. These included Cudahy et al. [25] in HIV-associated MDR-TB patients (aHR 2.94, 95% CI: 1.03–8.35), Kobayashi et al. [20] and Wang et al. [22] in hospitalized pulmonary TB, Komiya et al. [21] in elderly patients (aOR 1.057, 95% CI: 1.014–1.101), and Liu et al. [9] in ICU-admitted patients (aOR 1.012, 95% CI: 1.004–1.019),. In contrast, four studies did not find significant associations or reported only borderline significance. For example, Kim et al. (2012) [23] reported higher CRP levels in non-survivors than survivors in univariate analysis (mean 12.38 mg/dL vs. 6.95 mg/dL, *p* < 0.001), but the aOR was not significant (OR 1.62, 95% CI: 0.38–6.95, *p* = 0.517). Kan et al. (2019) [19] found a marginally non-significant association in univariate analysis (cHR 1.046, 95% CI: 0.997–1.097, *p* = 0.064) that was lost in multivariate models. Similarly, Huang et al. (2014) [24] reported higher CRP in non-survivors in univariate analysis (42.1 ± 59.4 mg/L vs. 12.5 ± 29.1 mg/L, *p* = 0.004), but CRP was excluded from the multivariate model. Honjo et al. (2020) [18] observed significance for TB-related death in univariate analysis (cHR 1.076, 95% CI: 1.026–1.127, *p* = 0.002) but not in competing risk regression (aHR 0.990, 95% CI: 0.916–1.070, *p* = 0.804). Non-significant findings were often attributed to small sample sizes, wide confidence intervals, stronger competing prognostic factors such as albumin or performance status, retrospective study designs, and heterogeneous definitions of mortality.

Outcome measures differed according to study design. Cohort studies typically reported hazard ratios derived from Cox regression, whereas case-control and hospital-based studies used odds ratios for dichotomous outcomes. Adjusted odds ratios (aORs) from three studies varied widely. Liu et al. (2020) [9] and Wang et al. (2019) [22] reported statistically significant aORs of 1.012 and 1.10, respectively. In contrast, Kim et al. (2012) [23] reported a higher aOR of 1.62, but this value was not statistically significant. While adjusted hazard ratios ranged from 1.04 in Kobayashi et al. (2024) [20] to 2.94 in Cudahy et al. (2018) [25], with intermediate estimates such as 1.25 in Huang et al. (2014) [24] not significant. Collectively, significant associations were observed more frequently in specific pulmonary TB populations, particularly among hospitalized patients, HIV co-infected patients, or ICU-admitted patients. As for elderly patients, Komiya et al. [21] found a significant association, however Honjo et al. [18] only found a significant association in the crude values, but not correlated after adjusting for confounders. These findings suggest that CRP may be more prognostic in severely ill or immunocompromised populations rather than in the general TB population.

### Pooled analysis according to effect estimate type

**Fig. 2:**
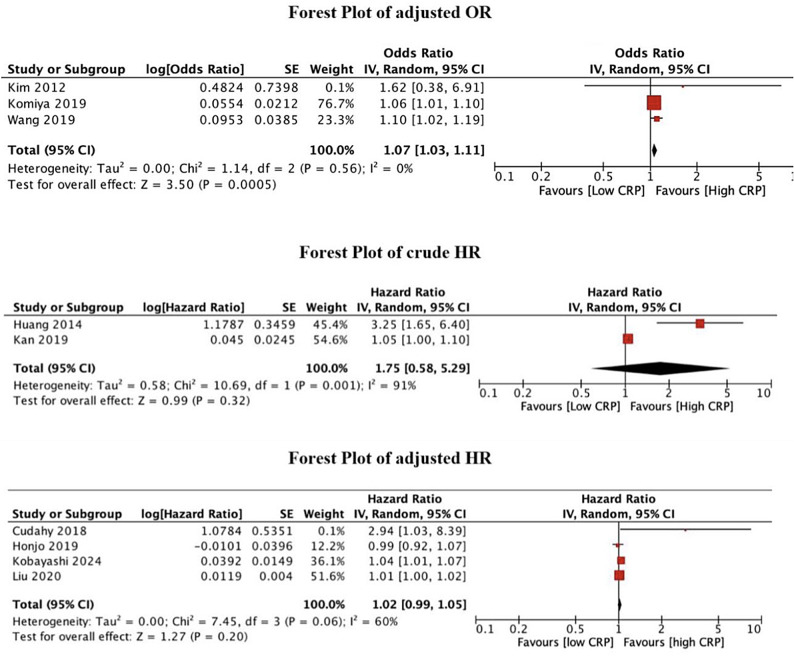
Forest plot of the studies

As the study designs varied across included studies, data synthesis was classified into three groups based on reported effect size: adjusted odds ratios (aOR), crude hazard ratios (cHR), and adjusted hazard ratios (aHR) (Fig. 2). Planned subgroup meta-analyses could only be performed based on CRP reporting because no other subgroup classification, either age-based population, HIV status, or TB subtype, contained more than one study reporting compatible effect estimates. While pooled analysis could be done for studies reporting CRP as continuous indicator and aOR as their effect size, similar analysis based on dichotomized CRP was not feasible because only two studies reported dichotomized CRP and the effect measures were not comparable. In addition, all studies with aHR as their effect size reported CRP as continuous indicator, so relevant subgroup analysis wasn’t needed.

Three studies reported aORs for the association between baseline CRP and mortality in TB patients [21–23]. Pooled analysis showed an overall aOR of 1.07 (95% CI 1.03–1.11; *p* = 0.0005), suggesting a statistically significant association. Heterogeneity was very low (I² = 0%). In a subgroup restricted to studies treating CRP as a continuous variable, the association remained significant (aOR 1.10; 95% CI 1.02–1.19; *p* = 0.01), with similarly low heterogeneity (I² = 0%) as seen in Appendix 7.

Another two studies reported crude HRs without adjustment for confounders [19, 24]. The pooled cHR was 1.75 (95% CI 0.58–5.29; *p* = 0.32), indicating no statistically significant association, although the point estimate suggested a higher risk. Heterogeneity between these studies was very high (I² = 91%, χ² = 10.69, *p* = 0.001), reflecting considerable inconsistency.

Meanwhile, four studies reported aHRs for baseline CRP and mortality in TB patients [9, 18, 20, 25]. After pooling, there was no statistically significant association (aHR 1.02, 95% CI 0.99–1.05; *p* = 0.20). Heterogeneity was moderate (I² = 60%, χ² = 7.45, *p* = 0.06), largely driven by one study in which Cudahy et al. reported an almost threefold higher risk of mortality (HR 2.94, 95% CI 1.03–8.39), whereas other studies reported a lower adjusted effects close to unity. While sensitivity analysis (Appendix 6) was conducted by removing the study by Cudahy due to low sample size and serial CRP level measurement, the heterogeneity was reduced to 47%. However, it was still considerable as a high heterogeneity and the result was still insignificant (aHR 1.02; 95% CI: 1.00-1.04; *p* = 0.12).

Overall, crude estimates tended to be stronger than adjusted estimates, suggesting confounding by factors such as age, comorbidities, disease severity, or drug resistance. The variability observed, particularly across HR-based analyses, likely reflects differences in study populations, settings, outcome definitions, CRP assay methods, and covariate adjustment strategies.

## Discussions

Here, we reviewed and evaluated the performance of baseline CRP level as a prognostic biomarker for mortality in adults with TB. Generally, we found that baseline CRP alone was not significantly associated with high risk of death in TB patients when considering either the aHR or cHR, although crude risk analyses showed a higher tendency to cause death than adjusted analyses (Fig. 2). The discrepancy between crude and adjusted results suggests that the apparent prognostic impact of CRP is at least partly confounded by underlying disease severity, comorbidities, or demographic factors, which are themselves strong determinants of mortality in tuberculosis. The pooled analysis for adjusted OR showed a significant result (OR = 1.07) with low heterogeneity, which means the chance of mortality increased slightly 7% in the high CRP group. A subgroup analysis including only studies with CRP reported as continuous indicator had a significant result (OR = 1.10).

Physiologically, CRP is an acute-phase protein produced by the liver in response to systemic inflammation, mainly through IL-6-mediated pathways [26, 27]. Unlike a pathogen-specific test, CRP is a marker of systemic inflammation that is elevated in response to a wide range of infectious and non-infectious conditions, including HIV and malignancies [5, 27, 28]. In patients with HIV, who are prone to opportunistic infection and often co-infected with TB, CRP levels were also elevated due to inflammation, further complicating the interpretation of high CRP as a mortality predictor biomarker [5]. Its elevation is ubiquitous in active TB and is influenced by a multitude of factors that themselves are direct drivers of mortality.

The majority of studies reported a nonsignificant correlation between CRP level and mortality, especially after adjusting for confounding factors. Some studies reported significant results; however, the hazard or chance of mortality was only a slight increase. It was reflected in our pooled analyses, where only the pooled analysis for adjusted OR showed a significant result, with only a slight increase in chance of mortality.

Here, we highlighted the explanation for why CRP has a low association with mortality. The most compelling evidence was the presence of confounders, supported by the fact that the risk was nullified following adjustment [18–23]. For instance, patients with more severe pulmonary TB or critical illness may have higher CRP levels and a greater risk of death [9]. In addition, patients with higher CRP often also had other indicators of severe illness, such as hypoxemia or critical disease. Comorbidities including diabetes mellitus and chronic kidney disease are themselves associated with worse TB outcomes and may contribute to both elevated inflammatory markers and higher mortality risk [29, 30]. In cases like these, death in a TB patient might be caused by the complication rather than the CRP level alone. Therefore, a high CRP level often simply reflects the presence of these established risk factors or the systemic inflammation response rather than providing unique prognostic information [18–23].

Significant heterogeneity among studies underscores challenges in defining CRP’s prognostic role. The substantial heterogeneity across studies further highlights the methodological variability in CRP measurement, including differences in cut-off thresholds, timing of sampling, and outcome definitions. Variability in patient populations, particularly regarding HIV co-infection or other comorbidities, further contributes to the high heterogeneity, leading to inconsistent findings. For example, Cudahy et al. reported an almost threefold increased mortality risk associated with elevated CRP, contrasting with other studies that found minimal or no effect [25]. The sensitivity analysis excluding study with small sample size and difference CRP testing method also showed a similar result, which was insignificant and high heterogeneity. Such discordance aligns with prior meta-analyses in infectious diseases, emphasizing the need for standardized biomarker assessment and uniform outcome definitions.

Our findings indicated that CRP might not be a valuable independent prognostic marker for TB mortality. However, increased baseline CRP has a tendency to be associated with mortality in certain cases, especially in elderly patients, HIV co-infected patients, hospitalized patients, or those admitted to the intensive care unit. Determination of baseline CRP in TB patients remains a useful marker for screening possible systemic inflammation that can be used for decision-making, together with other determining factors. Future research should aim to standardize CRP thresholds and integrate CRP into multivariable prognostic models that include clinical, radiological, and microbiological parameters. Combining CRP with other inflammatory and immunological biomarkers may also provide greater prognostic accuracy than CRP alone.

### Strengths, limitations, and implications

Although CRP is not a novel biomarker, its potential role as an accessible prognostic factor in TB remains clinically important, particularly in resource-constrained settings where more complex prognostic tools may not be readily available. The value of this review lies in systematically synthesizing the available evidence and clarifying the extent to which baseline CRP shows a consistent association with mortality across heterogeneous study populations and analytical approaches. Its strengths include adherence to standard systematic review methodology, the use of multiple appraisal frameworks (QUIPS and GRADE) for robust quality assessment, and the comprehensive inclusion of diverse study designs. By assessing both crude and adjusted effect estimates, this review provided a balanced evaluation of CRP’s apparent prognostic role while accounting for confounding factors [11, 14, 15].

Despite these strengths, important limitations were noted. The certainty of the evidence was rated as low to very low due to the reliance on observational studies, modest sample sizes, and significant methodological heterogeneity across studies. In addition, this review was restricted to English-language full-text articles, which may have introduced language bias and may have excluded relevant evidence published in other languages or not accessible in full text. Variability in CRP measurement methods, assay thresholds, and definitions of mortality limited comparability, and missing covariate data further restricted the ability to draw firm conclusions. However, available evidence gave little information regarding those covariates, which made subgroup analysis for these aspects not possible.

Given these limitations, this review does not provide sufficient evidence to support CRP as a stand-alone prognostic biomarker for mortality in pulmonary TB. However, its consistent association with worse outcomes in high-risk subgroups suggests that CRP may still be useful for prognostic stratification when integrated into multimodal models. Future research should prioritize large-scale prospective studies with standardized CRP protocols, uniform outcome definitions, and rigorous adjustment for key confounders. Additionally, combining CRP with other inflammatory markers, genetic predictors, or advanced imaging may enhance its predictive performance. Serial CRP measurements during treatment also warrant further investigation, as they may more accurately reflect treatment response than a single baseline value [7, 31]. Future research should regularly assess and collect data on the outcome of specific patient characteristics that may be related to higher risk of mortality, such as having diabetes mellitus, chronic kidney disease, advanced-TB disease, and older age, as there is still no sufficient data to conclude the relationship between a specific subgroup of TB patient, CRP level and its outcome.

## Conclusion

This review suggests that elevated baseline CRP was commonly observed in TB, although baseline CRP elevation appears largely confounded by disease severity and other factors, limiting its value as an independent prognostic biomarker. Nonetheless, baseline CRP evaluation might assist in systemic inflammation identification and clinical decision-making in high-risk populations, particularly elderly individuals, those with HIV co-infection, or critically ill populations. In terms of prognosis prediction, a comprehensive approach should integrate baseline CRP with clinical presentation and other biomarkers to improve accuracy.

## Electronic Supplementary Material

Below is the link to the electronic supplementary material.

Supplementary Material 1

## Data Availability

Complete data is available upon request to the authors.

## Declarations

### Supplementary information

The full systematic review protocol for this study is available through its registration on the International Prospective Register of Systematic Reviews (PROSPERO) under registration number CRD420251101984, and can also be accessed via DOI: 10.2196/preprints.80744. The supplementary Information can be assessed in the supplementary material file.

### Registration and protocol

The systematic review protocol for this study, titled “Prognostic Performance of C-reactive Protein for Tuberculosis Outcome,” was prospectively registered on the International Prospective Register of Systematic Reviews (PROSPERO). The registration number is CRD420251101984. The full protocol is publicly accessible.

### Use of generative AI

ChatGPT 5.2 (OpenAI) was used during manuscript preparation for language refinement and editorial assistance. All outputs were reviewed, revised, and verified by the authors, who take full responsibility for the final content.

## Abbreviations

aHR: Adjusted Hazard Ratio
aOR: Adjusted Odds Ratio
AUC: Area Under the Curve
CHARMS-PF: Critical Appraisal and Data Extraction for Systematic Reviews of Prediction Modelling Studies for Prognostic Factors
cHR: Crude Hazard Ratio
CI: Confidence Interval
CRP: C-reactive Protein
GRADE: Grading of Recommendations Assessment, Development, and Evaluation
HIV: Human Immunodeficiency Virus
ICU: Intensive Care Unit
IQR: Interquartile Range
MDR-TB: Multidrug-Resistant Tuberculosis
PRISMA: Preferred Reporting Items for Systematic Reviews and Meta-Analyses
PROSPERO: International Prospective Register of Systematic Reviews
QUIPS: Quality in Prognosis Studies
RCT: Randomized Controlled Trial
TB: Tuberculosis
WHO: World Health Organization
