# Supplementary material for: C-reactive protein as a predictor of mortality in tuberculosis: systematic review and meta-analysis
Source: BMC Infect Dis. 2026 May 6;26:1205. doi: 10.1186/s12879-026-13462-9 (PMC13325576; doi:10.1186/s12879-026-13462-9)

**Appendix**

**Appendix 1.**

PRISMA 2020 Checklist

| **Section and Topic** | **Item #** | **Checklist item** | **Location where item is reported** |
| --- | --- | --- | --- |
| **TITLE** | | |  |
| Title | 1 | Identify the report as a systematic review. | 1 |
| **ABSTRACT** | | |  |
| Abstract | 2 | See the PRISMA 2020 for Abstracts checklist. | 1 |
| **INTRODUCTION** | | |  |
| Rationale | 3 | Describe the rationale for the review in the context of existing knowledge. | 3 |
| Objectives | 4 | Provide an explicit statement of the objective(s) or question(s) the review addresses. | 3 |
| **METHODS** | | |  |
| Eligibility criteria | 5 | Specify the inclusion and exclusion criteria for the review and how studies were grouped for the syntheses. | 4 |
| Information sources | 6 | Specify all databases, registers, websites, organisations, reference lists and other sources searched or consulted to identify studies. Specify the date when each source was last searched or consulted. | 5 |
| Search strategy | 7 | Present the full search strategies for all databases, registers and websites, including any filters and limits used. | 5 |
| Selection process | 8 | Specify the methods used to decide whether a study met the inclusion criteria of the review, including how many reviewers screened each record and each report retrieved, whether they worked independently, and if applicable, details of automation tools used in the process. | 7 |
| Data collection process | 9 | Specify the methods used to collect data from reports, including how many reviewers collected data from each report, whether they worked independently, any processes for obtaining or confirming data from study investigators, and if applicable, details of automation tools used in the process. | 8 |
| Data items | 10a | List and define all outcomes for which data were sought. Specify whether all results that were compatible with each outcome domain in each study were sought (e.g. for all measures, time points, analyses), and if not, the methods used to decide which results to collect. | 8 |
|  | 10b | List and define all other variables for which data were sought (e.g. participant and intervention characteristics, funding sources). Describe any assumptions made about any missing or unclear information. | 20 |
| Study risk of bias assessment | 11 | Specify the methods used to assess risk of bias in the included studies, including details of the tool(s) used, how many reviewers assessed each study and whether they worked independently, and if applicable, details of automation tools used in the process. | 8 |
| Effect measures | 12 | Specify for each outcome the effect measure(s) (e.g. risk ratio, mean difference) used in the synthesis or presentation of results. | 9 |
| Synthesis methods | 13a | Describe the processes used to decide which studies were eligible for each synthesis (e.g. tabulating the study intervention characteristics and comparing against the planned groups for each synthesis (item #5)). | 9 |
|  | 13b | Describe any methods required to prepare the data for presentation or synthesis, such as handling of missing summary statistics, or data conversions. | 9 |
|  | 13c | Describe any methods used to tabulate or visually display results of individual studies and syntheses. | 9 |
|  | 13d | Describe any methods used to synthesize results and provide a rationale for the choice(s). If meta-analysis was performed, describe the model(s), method(s) to identify the presence and extent of statistical heterogeneity, and software package(s) used. | 9 |
|  | 13e | Describe any methods used to explore possible causes of heterogeneity among study results (e.g. subgroup analysis, meta-regression). | 9 |
|  | 13f | Describe any sensitivity analyses conducted to assess robustness of the synthesized results. | 9 |
| Reporting bias assessment | 14 | Describe any methods used to assess risk of bias due to missing results in a synthesis (arising from reporting biases). | 9 |
| Certainty assessment | 15 | Describe any methods used to assess certainty (or confidence) in the body of evidence for an outcome. | 10 |
| **RESULTS** | | |  |
| Study selection | 16a | Describe the results of the search and selection process, from the number of records identified in the search to the number of studies included in the review, ideally using a flow diagram. | 10 |
|  | 16b | Cite studies that might appear to meet the inclusion criteria, but which were excluded, and explain why they were excluded. | 10 |
| Study characteristics | 17 | Cite each included study and present its characteristics. | 10 |
| Risk of bias in studies | 18 | Present assessments of risk of bias for each included study. | 10 |
| Results of individual studies | 19 | For all outcomes, present, for each study: (a) summary statistics for each group (where appropriate) and (b) an effect estimate and its precision (e.g. confidence/credible interval), ideally using structured tables or plots. | 15 |
| Results of syntheses | 20a | For each synthesis, briefly summarise the characteristics and risk of bias among contributing studies. | 15 |
|  | 20b | Present results of all statistical syntheses conducted. If meta-analysis was done, present for each the summary estimate and its precision (e.g. confidence/credible interval) and measures of statistical heterogeneity. If comparing groups, describe the direction of the effect. | 15 |
|  | 20c | Present results of all investigations of possible causes of heterogeneity among study results. | 15 |
|  | 20d | Present results of all sensitivity analyses conducted to assess the robustness of the synthesized results. | 15 |
| Reporting biases | 21 | Present assessments of risk of bias due to missing results (arising from reporting biases) for each synthesis assessed. | 17 |
| Certainty of evidence | 22 | Present assessments of certainty (or confidence) in the body of evidence for each outcome assessed. | 17 |
| **DISCUSSION** | | |  |
| Discussion | 23a | Provide a general interpretation of the results in the context of other evidence. | 17 |
|  | 23b | Discuss any limitations of the evidence included in the review. | 18 |
|  | 23c | Discuss any limitations of the review processes used. | 18 |
|  | 23d | Discuss implications of the results for practice, policy, and future research. | 19 |
| **OTHER INFORMATION** | | |  |
| Registration and protocol | 24a | Provide registration information for the review, including register name and registration number, or state that the review was not registered. | 20 |
|  | 24b | Indicate where the review protocol can be accessed, or state that a protocol was not prepared. | 20 |
|  | 24c | Describe and explain any amendments to information provided at registration or in the protocol. | 20 |
| Support | 25 | Describe sources of financial or non-financial support for the review, and the role of the funders or sponsors in the review. | 22 |
| Competing interests | 26 | Declare any competing interests of review authors. | 23 |
| Availability of data, code and other materials | 27 | Report which of the following are publicly available and where they can be found: template data collection forms; data extracted from included studies; data used for all analyses; analytic code; any other materials used in the review. | 22 |

**Appendix 2.**

PRISMA 2020 Abstract Checklist

| **Section and Topic** | **Item #** | **Checklist item** | **Reported (Yes/No)** |
| --- | --- | --- | --- |
| **TITLE** | | |  |
| Title | 1 | Identify the report as a systematic review. | Yes |
| **BACKGROUND** | | |  |
| Objectives | 2 | Provide an explicit statement of the main objective(s) or question(s) the review addresses. | Yes |
| **METHODS** | | |  |
| Eligibility criteria | 3 | Specify the inclusion and exclusion criteria for the review. | Yes |
| Information sources | 4 | Specify the information sources (e.g. databases, registers) used to identify studies and the date when each was last searched. | Yes |
| Risk of bias | 5 | Specify the methods used to assess risk of bias in the included studies. | Yes |
| Synthesis of results | 6 | Specify the methods used to present and synthesise results. | Yes |
| **RESULTS** | | |  |
| Included studies | 7 | Give the total number of included studies and participants and summarise relevant characteristics of studies. | Yes |
| Synthesis of results | 8 | Present results for main outcomes, preferably indicating the number of included studies and participants for each. If meta-analysis was done, report the summary estimate and confidence/credible interval. If comparing groups, indicate the direction of the effect (i.e. which group is favoured). | Yes |
| **DISCUSSION** | | |  |
| Limitations of evidence | 9 | Provide a brief summary of the limitations of the evidence included in the review (e.g. study risk of bias, inconsistency and imprecision). | Yes |
| Interpretation | 10 | Provide a general interpretation of the results and important implications. | Yes |
| **OTHER** | | |  |
| Funding | 11 | Specify the primary source of funding for the review. | Yes |
| Registration | 12 | Provide the register name and registration number. | Yes |

**Appendix 3.**

**Table 1.** Search Strategy - PubMed

DATE: 10/07/25; Retrieved data: 516

| Query # | Query Terms |
| --- | --- |
| 1 | (“tuberculosis” OR “TB”) |
| 2 | (“C-reactive protein” OR “C reactive protein” OR “CRP”) |
| 3 | (“prognosis” OR “mortality” OR “death” OR “survival analysis”) |
| 4 | #1 AND #2 AND #3 |

**Table 2.** Search Strategy - Scopus

DATE: 10/07/25; Retrieved data: 283

| Query # | Query Terms |
| --- | --- |
| 1 | TITLE-ABS (“tuberculosis” OR “TB) |
| 2 | TITLE-ABS (“C-reactive protein” OR “C reactive protein” OR “CRP”) |
| 3 | TITLE-ABS (“mortality” OR “death” OR “prognosis” OR “survival analysis”) |
| 4 | #1 AND #2 AND #3 |

**Table 3.** Search Strategy - Cochrane Library

DATE: 10/07/25; Retrieved data: 0

| Query # | Query Terms |
| --- | --- |
| 1 | (“tuberculosis” OR “TB”):ti,ab |
| 2 | “C-reactive protein” OR “C reactive protein” OR “CRP”):ti,ab |
| 3 | (“mortality” OR “death” OR “prognosis” OR “survival analysis”):ti,ab |
| 4 | #1 AND #2 AND #3 |

**Table 4.** Search Strategy - MEDLINE (via Ovid)

DATE: 10/07/25; Retrieved data: 89

| Query # | Query Terms |
| --- | --- |
| 1 | exp Tuberculosis/ OR tuberculosis.tw. |
| 2 | exp C-Reactive Protein/ OR CRP.tw. |
| 3 | exp Mortality/ OR death.tw. OR prognosis.tw. OR survival.tw. |
| 4 | #1 AND #2 AND #3 |

Table 5. Search Strategy - Proquest

DATE: 10/07/25; Retrieved data: 92

| Query # | Query Terms |
| --- | --- |
| 1 | (ti(tuberculosis) OR ti(TB) OR ab(tuberculosis) OR ab(TB)) |
| 2 | (ti("C-reactive protein") OR ti("C reactive protein") OR ti(CRP) OR ab("C-reactive protein") OR ab("C reactive protein") OR ab(CRP)) |
| 3 | ti(mortality) OR ti(death) OR ti(prognosis) OR ti("survival analysis") OR ab(mortality) OR ab(death) OR ab(prognosis) OR ab("survival analysis")) |
| 4 | #1 AND #2 AND #3 |

Table 6. Search Strategy - medRxiv

**DATE: 14/07/25; Retrieved data: 297**

| Query # | Query Terms |
| --- | --- |
| 1 | (tuberculosis OR TB) AND ("c reactive protein" OR CRP) AND (mortality OR death OR prognosis OR "survival analysis") |

**Appendix 4.**

Several studies that initially appeared to meet the inclusion criteria were excluded after the abstract screening process. These studies are excluded (table 9):

**Table 1.** Studies that might appear to meet the inclusion criteria, but which were excluded.

| **No.** | **Title** | **Author** | **Excluded reason** |
| --- | --- | --- | --- |
| 1 | A Scoring System Based on Laboratory Parameters and Clinical Features to Predict Unfavorable Treatment Outcomes in Multidrug-and Rifampicin-Resistant Tuberculosis Patients | Yan et al. | Wrong determinant (composite score CPR) and specific MDR-TB population |
| 2 | Associations of Hyponatremia and SIADH with Increased Mortality, Young Age and Infection Parameters in Patients with Tuberculosis | Bal et al. | Wrong determinant; Primarily examined hyponatremia, not CRP levels as prognostic determinant |
| 3 | Lung and Blood Early Biomarkers for Host-Directed TB Therapies: Secondary Outcome Measures from a Randomized Controlled Trial | Wallis et al. | Wrong outcome/determinant; CRP not assessed for mortality |
| 4 | Evaluation of Tuberculosis Treatment Response With Serial C-Reactive Protein Measurements | Wilson et al. | Wrong outcome; focuses on treatment response, not mortality |
| 5 | Incidence, Clinical Spectrum, Risk Factors and Impact of HIV-Associated Immune Reconstitution Inflammatory Syndrome in South Africa | Haddow et al. | Wrong domain; HIV-associated IRIS, not general TB mortality |
| 6 | Iron Status Predicts Treatment Failure and Mortality in Tuberculosis Patients: A Prospective Cohort Study from Dar es Salaam, Tanzania | Isanaka et al. | Wrong determinant; Focused on iron status, not CRP levels as determinant |
| 7 | Risk Factors for Mortality in Patients with Tuberculosis Admitted to Intensive Care Units | Wang et al. | Wrong determinant/outcome; CRP not analyzed |
| 8 | Predictors on In-Hospital Mortality Following In-Hospital Diagnosis of Tuberculosis | Shin et al. | Foreign Language |
| 9 | Clinical Characteristics and Prognostic Factors in Patients with Pulmonary Tuberculosis Admitted to Intensive Care Units | Han et al. | Foreign Language |
| 10 | Tuberculosis in Hemodialysis | El Amrani et al. | Foreign Language |
| 11 | Biomarkers of Mortality in Adults and Adolescents with Advanced HIV in Sub-Saharan Africa | Riitho et al. | Wrong population; adolescents subject |
| 12 | Predictive Value of C-Reactive Protein for Tuberculosis, Bloodstream Infection or Death Among HIV-Infected Individuals with Chronic, Non-Specific Symptoms and Negative Sputum Smear Microscopy | Bedell et al. | Wrong outcome (composite); wrong population (HIV + symptoms) |
| 13 | Impact of Anemia on Prognosis in Tuberculosis Patients | Luo et al. | Wrong determinant; CRP secondary to anemia analysis |
| 14 | High-Sensitivity C-Reactive Protein in HIV Care: Tuberculosis Diagnosis and Short-Term Mortality in a Cohort of Kenyan HIV Patients in the DREAM Programme | Ciccacci et al. | Wrong domain/determinant; HIV focus, CRP used in composite |
| 15 | C-Reactive Protein, Neopterin and Beta2 Microglobulin Levels Pre and Post TB Treatment in The Gambia | Mendy et al. | Wrong outcome; no mortality analysis |
| 16 | Mortality and Predictors in Pulmonary Tuberculosis with Respiratory Failure Requiring Mechanical Ventilation | Kim et al. | Wrong domain; highly specific ICU/mechanical ventilation group |
| 17 | Elderly Patients with Tuberculosis in a Low-Incidence Country - Clinical Characteristics, Inflammation and Outcome | van Arkel et al. | Wrong domain; No quantitative linkage |
| 18 | Plasma Levels of CRP, Neopterin and IP-10 in HIV-Infected Individuals with and without Pulmonary Tuberculosis | Ciccacci et al. | Mixed population, wrong determinant (multiple markers) |
| 19 | The Association of Atherosclerotic Cardiovascular Disease and Statin Use with Inflammation and Treatment Outcomes in Tuberculosis | Chidambaram et al. | Wrong determinant; CRP used as inflammation proxy |
| 20 | Serum Procalcitonin in Pulmonary Tuberculosis | Rasmussen et al. | Wrong determinant; CRP not the main prognostic factor |
| 21 | Clinical Characteristics of Nursing- and Healthcare-Associated Tuberculosis | Suzuki et al. | Wrong domain; lack of determinant/outcome focus; no CRP or mortality focus |
| 22 | Characteristics and Clinical Outcome of Bone and Joint Tuberculosis From 1994 to 2011: A Retrospective Register-Based Study in Denmark | Johansen et al. | Wrong domain; lack of determinant/outcome focus; no mortality/CRP linkage |
| 23 | Diagnostic and Prognostic Value of Serum C-Reactive Protein for Screening for HIV-Associated Tuberculosis | Lawn et al. | Wrong outcome; no quantitative CRP-mortality analysis |
| 24 | Higher Serum Cholesterol Levels Are Associated with Reduced Systemic Inflammation and Mortality During Tuberculosis Treatment Independent of Body Mass Index | Chidambaram et al. | Wrong determinant; cholesterol is primary focus |
| 25 | Adult Tuberculous Meningitis in Qatar: A Descriptive Retrospective Study from Its Referral Center | Imam et al. | Wrong domain; CNS TB, no CRP mortality analysis |
| 26 | Tuberculosis in Patients with End-Stage Renal Disease | Andrew OT et al. | Wrong domain; ESRD-specific, no CRP outcome data |
| 27 | Clinical Significance of Glasgow Prognostic Score in Patients with Tuberculous Pleurisy | Kang et al. | wrong determinant (composite score) and specific domain; primary determinant is GPS not CRP |
| 28 | Prognostic Values of Serum IP-10 and IL-17 in Patients with Pulmonary Tuberculosis | Chen et al. | Wrong determinant; CRP not assessed as determinant |
| 29 | Risk Factors for Mortality Among Malnourished HIV-Infected Adults Eligible for Antiretroviral Therapy | Woodd et al. | Wrong domain; HIV-malnutrition focus, not TB-specific |
| 30 | Extrapulmonary Tuberculosis: 7 Year-Experience of a Tertiary Center in Istanbul | Sevgiet al. | Wrong domain; extrapulmonary TB only, no CRP analysis |
| 31 | Characteristics of Patients with Spinal Tuberculosis: Seven-Year Experience of a Teaching Hospital in Southwest China | Wang et al. | Wrong domain; spinal TB only, no CRP-mortality data |
| 32 | A Nomogram for Predicting Unfavorable Outcomes of Antituberculosis Treatment Among Individuals with AIDS Combined with Pulmonary Tuberculosis in China | Han et al. | Wrong determinant (CAR), and specific AIDS+TB population |
| 33 | Characteristics and predictors for tuberculosis related mortality in Denmark from 2009 through 2014: A retrospective cohort study | Holden et al. | Wrong population; there is pediatric population |
| 34 | Concomitant Pulmonary Tuberculosis and Lung Cancer: A Case-Control Study of Risk and Prognostic Factors | Zegmout et al. | Wrong outcome; there is no quantitative CRP-mortality of tuberculosis measurement |
| 35 | Biomarkers Associated with Death After Initiating Treatment for Tuberculosis and HIV in Patients with Very Low CD (4) Cells | Sattle al. | Wrong population; there is clinical tested TB in the study |

**Appendix 5**

**Table 1** Risk of bias and GRADE assessment of included studies.

| **Study** | **Design** | **Risk of Bias Assessment** | **Missing Data** | **Outcome Assessed** | **Certainty (GRADE)** | **Interpretation (based on GRADE domains)** |
| --- | --- | --- | --- | --- | --- | --- |
| Cudahy (2018)[26] | Prospective cohort | QUIPS: Low Risk (most), Moderate (Study Participation) | Missing data were not explicitly reported. While participants were followed for 16 weeks with regular biomarker sampling, the handling of missed timepoints was not described. No imputation or sensitivity analyses were conducted. | 16-week mortality (HIV/MDR-TB) | ⨁◯◯◯Very Low | Serious imprecision (small sample, wide CI), indirectness (unique HIV/MDR-TB population), and risk of bias (moderate in study participation, incomplete accounting of data). |
| Kobayashi (2024)[27] | Retrospective case-control | QUIPS: Low Risk (all domains) | Patients with indeterminate or borderline T-SPOT.TB results were excluded, though these were reportedly rare. Other variables (e.g., CRP, NLR, albumin) were assumed complete, as data were reported for all 244 patients. However, unmeasured variables such as TB severity and treatment adherence were acknowledged as limitations. | In-hospital mortality | ⨁◯◯◯Very Low | Due to its retrospective design, imprecision, and risk of unmeasured confounding |
| Komiya (2020)[23] | Retrospective cohort | QUIPS: Low Risk (all domains) | Missing data were not reported for the primary outcome (sputum conversion). Some potential predictor variables lacked information, but handling methods were not described. | Mortality & sputum conversion | ⨁◯◯◯Very Low | Study limitations (risk of residual confounding), no missing data strategy, and observational design with risk of unmeasured bias. |
| Kan (2019)[24] | Retrospective cohort | QUIPS: Low Risk (all domains) | Missing data were not reported or discussed. | In-hospital mortality | ⨁◯◯◯Very Low | due to borderline statistical significance and the presence of multiple unmeasured confounders. |
| Liu (2020)[28] | Retrospective cohort | QUIPS: Low Risk (most), Moderate (statistical analysis and  reporting) | Patients with incomplete clinical data were excluded from analysis. However, the total number of excluded individuals was not reported, and no imputation methods were described. | ICU mortality | ⨁◯◯◯Very Low | Moderate risk of bias (statistical reporting), selection bias from excluding incomplete data, and indirectness due to limited external validity. |
| Honjo (2020)[25] | Retrospective cohort | QUIPS: Low Risk (all domains) | Missing data were not reported or discussed. | TB-related mortality | ⨁⨁◯◯Low | Observational design with complete follow-up, but imprecision in multivariable model and potential residual confounding kept certainty at low. |
| Kim et al. (2012)[29] | Case-control | QUIPS: Low Risk (all domains) | Missing data were not reported or discussed. | All-cause mortality | ⨁◯◯◯Very Low | Small case-control design, imprecision (non-significant adjusted model), and study limitations (recall/selection bias, missing cause of death). |
| Huang (2014)[30] | Prospective cohort | QUIPS: Low Risk (all domains) | The authors did not mention missing data or describe any imputation methods, suggesting possible complete case analysis, but this remains unclear. | 6-month mortality | ⨁⨁◯◯Low | Prospective design improves confidence, but residual confounding and non-significant adjusted models limit certainty. |
| Wang (2019)[31] | Prospective cohort | QUIPS: Low Risk (all domains) | Missing data were not reported or discussed. | 12-month all-cause mortality | ⨁⨁◯◯Low | Observational study with no major flaws, but default risk of bias (confounding) and no upgrading factors keep certainty at low. |

**Appendix 6.** Sensitivity Analysis for Adjusted HR


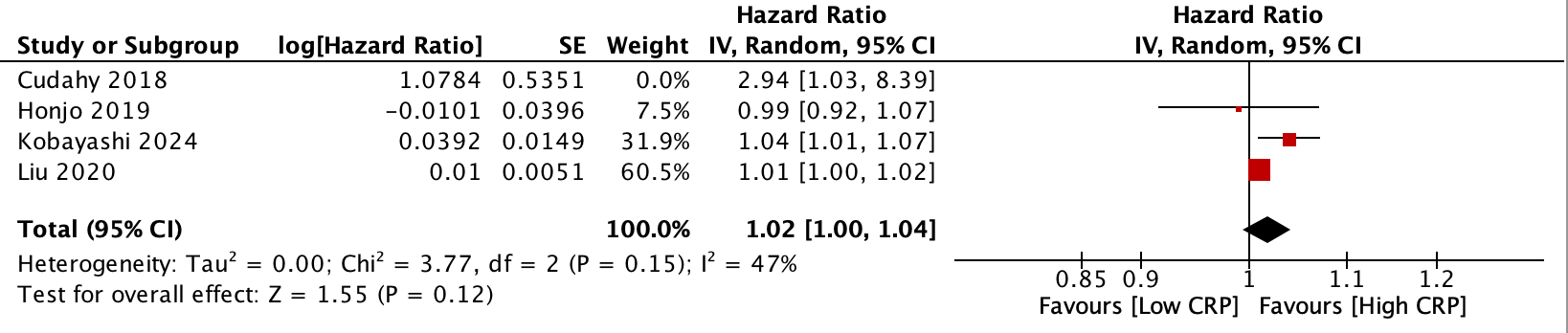


**Appendix 7.** Sensitivity Analysis for Adjusted OR for Continuous CRP Value


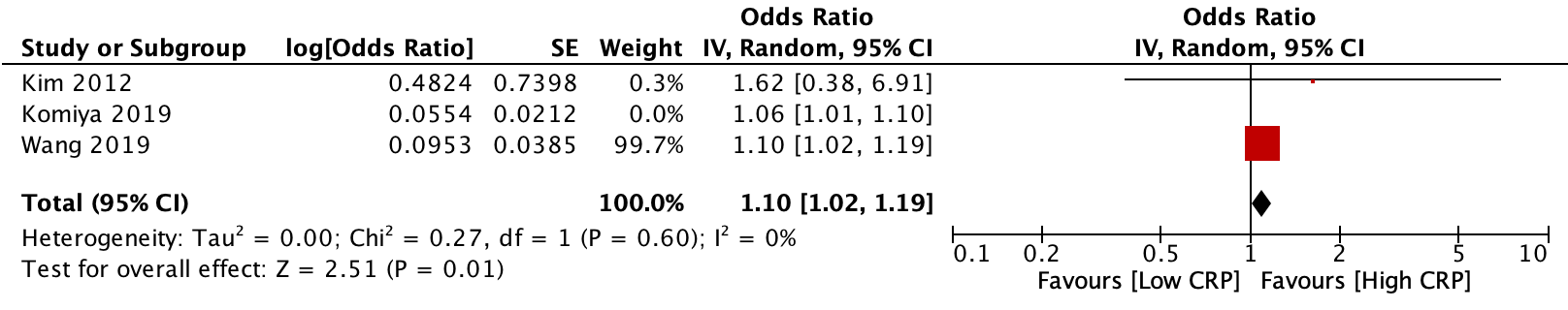

Supplement: Supplementary file 1 — Supplementary Material 1 [file 12879_2026_13462_MOESM1_ESM.docx]
